# Selenium Accumulation, Antioxidant Enzyme Levels, and Amino Acids Composition in Chinese Mitten Crab (*Eriocheir sinensis*) Fed Selenium-Biofortified Corn

**DOI:** 10.3390/nu10030318

**Published:** 2018-03-07

**Authors:** Linxi Yuan, Ru Zhang, Xuzhou Ma, Ling Yang, Qing Zheng, Dong Chen, Miao Li, Ting Fan, Yongxian Liu, Liping Pan, Xuebin Yin

**Affiliations:** 1 Jiangsu Bio-Engineering Research Center of Selenium, Suzhou 215123, China; yuanlinxi001@gmail.com (L.Y.); zhangru1108@163.com (R.Z.); zheng.q@setek.com (Q.Z.); chen.d@setek.com (D.C.); 2 School of Resources and Environment, Anhui Agricultural University, Hefei 230036, China; plantprotection2006@126.com (M.L.); fanting@ahau.edu.cn (T.F.); 3 College of Fisheries and Life Sciences, Shanghai Ocean University, Shanghai 201306, China; xzma@shou.edu.cn; 4 Suzhou Polytechnic Institute of Agriculture, Suzhou 215008, China; yanglingnj@sina.com; 5 Institute of Agricultural Resource and Environment, Guangxi Academy of Agricultural Sciences, Nanning 530007, China; liuyx27@163.com (Y.L.); plping1013@163.com (L.P.); 6 School of Earth and Space Sciences, University of Science and Technology of China, Hefei 230026, China

**Keywords:** Se-biofortified corn, Chinese mitten crab, muscle Se level, hemolymph supernatant, antioxidant levels, amino acids content

## Abstract

The effects of selenium (Se)-biofortified corn on the total Se contents, the antioxidant enzyme levels, and the amino acids composition in Chinese mitten crab (*Eriocheir sinensis*) during the stage of the fifth shelling to maturity were investigated in the present study. The culture density of crabs was 600 per 667 m^2^, and they were continuously fed 120.4 mg Se from Se-biofortified corn per 667 m^2^ every two days for 90 days. The results showed that the total muscle Se levels in the crabs were significantly increased (*p* < 0.05). Activities of hemolymph supernatant enzymes including alkaline phosphatase (AKP), lysozyme (LZM), glutathione peroxidase (GPx), and superoxide dismutase (SOD) were also enhanced (*p* < 0.05). The protein and crude fat levels at maturity were higher than those at the fourth molt. The levels of total essential amino acids (∑EAAs) and total delicious amino acids (∑DAAs) were significantly increased (*p* < 0.05). We demonstrate that the feeding of Se-biofortified corn could significantly improve total muscle Se concentrations and hemolymph supernatant antioxidant enzyme activities in Chinese mitten crab, and slow down the rapid decline of ∑EAAs and ∑DAAs at maturity, thus improving the nutritional quality of Chinese mitten crab.

## 1. Introduction

The aquaculture of Chinese mitten crab (*Eriocheir sinensis*) plays an important role in the freshwater fishery in China, with a total annual yield of 700,000 tons in 2012 [1]. It is generally believed that there are no immunoglobulins in Chinese mitten crab body fluids and the antibody-mediated immune response. The immune system of Chinese mitten crab consists of the phagocytosis and encapsulation by hemolymph cells; activities of enzymes such as alkaline phosphatases (AKP), lysozymes (LZM), glutathione peroxidase (GPx), and superoxide dismutase (SOD); activities of immune factors including lectins and hemolysin, etc.; and reactions of regulatory factors such as the thephenol oxidase-activating system [2]. Environmental stresses including water eutrophication and water pollution may lead to the low levels of dissolved oxygen, which along with the rapid growth of pathogenic bacteria especially in summer may increase the morbidity and mortality of Chinese mitten crab (*Eriocheir sinensis*) [3]. It is thus important to study ways to improve the anti-hypoxia ability as well as immunity of Chinese mitten crab in China.

As one of the essential nutrient elements, the proper addition of selenium (Se) may significantly improve the anti-hypoxia ability and immunity of aquatic creatures [4]. It has been suggested that Se can improve the growth and physiological performance of creatures including rats [5], fishes [6,7], and crustaceans [8,9]. Selenium also could help crustaceans increase immunity and disease resistance [10]. However, different sources of Se lead to different assimilation levels, and thus result in different effects on aquatic animals. Bell and Cowey (1989) found that the digestibility of selenomethionine (SeMet) was as high as 91.6% in Atlantic salmon, whereas that of sodium selenite (Na_2_SeO_3_) was 63.9% [11]. Wang et al. (1997) studied the effects of dietary Se on channel catfish (*Ictalurus punctatus*) to show that the organic Se had higher bioavailability than inorganic Se [12]. Le and Fort (2014) found that organic Se could produce more weight gain, higher Se accumulation in muscle tissues, and higher bactericidal activity than selenite in yellowtail kingfish (*Seriolalalandi*) [13]. Moreover, dietary SeMet increased the crude protein contents on the body wall of juvenile sea cucumber (*Apostichopus japonicus*) [14].

Biofortification technology has been widely applied in animals/agriculture [15,16,17,18]. The Se-biofortified agricultural products contain high levels of Se (e.g., 5–10 µg·kg^−1^ in corn) with SeMet and Se-methylselenocysteine (MeSeCys) as the major Se-containing compounds, thus being used as a new source of Se [18]. The Se-biofortified products might have potential benefits for animal nutrition worldwide [19]. The present study focused on the effects of Se-biofortified corn on the total body Se contents, the antioxidant enzyme levels, and the amino acids composition in Chinese mitten crab (*Eriocheir sinensis*) to evaluate the application of grain-biofortified Se in aquaculture.

## 2. Materials and Methods

### 2.1. Materials

Chinese mitten crabs (*Eriocheir sinensis*) (average larval weight: 6.25 g) were provided by Jiangxin Island Chinese Mitten Crab Aquaculture Base in Zhangjiagang, Suzhou, Jiangsu. Se-biofortified corn (total Se level as 5.35 mg·kg^−1^ with 60% SeMet) was provided by Suzhou Setek Co. Ltd., Suzhou, Jiangsu, China. Crab feedstuff was purchased from Jiangsu Jinkangda Group. Normal corn (with a total selenium level of 0.04 mg·kg^−1^) was purchased from the market.

### 2.2. Experiment Diets and Design

The experiments were carried out in eight aquaculture ponds with a size of about 13,340 m^2^ each in Jiangxin Island Chinese Mitten Crab Aquaculture Base in Zhangjiagang, Suzhou, Jiangsu Province. Six hundred per 667 m^2^ of the larval crabs were released into the aquaculture ponds on 7 March 2015, and harvested on 30 September 2015. Crabs were fed a diet with the crab feedstuff at 15 kg/day/667 m^2^ and normal corn once every two days at 1.5 kg/667 m^2^ each time. Right after the fourth shelling, crabs in four of the aquaculture ponds were fed crab feedstuff daily along with 0.5 kg of Se-biofortified corn plus 1.0 kg of normal corn per 667 m^2^ once every two days. The control group (CK) consisted of the crabs in the other four ponds, which were given the same diet as that used before the fourth shelling.

### 2.3. Sample Collection, Preparation, and Storage

Crab samples were collected on 29 July 2015 (right after the fourth shelling, as control groups (CK)), 30 August 2015 (right after the fifth shelling), and 30 September 2015 (at the harvest stage), respectively. Crab samples were randomly chosen and contained six male and six female crabs in each pond every time. Those crab samples were transported with ice to the lab to collect the hemolymph and meat samples within 3 h.

For each crab, 8 mL hemolymph was collected by leg shearing and centrifuged (3500 rpm, 4 °C and 10 min). The supernatant was stored at −80 °C. The crabmeat sample was taken from the legs and the abdomen and stored at −20 °C after being smashed by a grinder.

Since no significant difference was found between the male and the female when detecting the total Se levels in crabmeat, the samples were no longer separated according to gender for supernatant enzymes, amino acids, proteins, and fats measurement so as to reduce cost.

### 2.4. Determination of the Total Selenium Level

About 0.5 g of the crabmeat sample was accurately quantified, put into an Erlenmeyer flask, and combined with 10 mL of a mixed acid (nitric acid and hydrochloric acid, with a volume ratio of 4:1). After more than 3 h of digestion at room temperature, the flask was heated for 1 h at 110 °C, 2 h at 130 °C, 1 h at 180 °C, and then at 210 °C till clouds of white smoke were produced and the volume of the solution was reduced to about 2 mL. After cooling to room temperature, the funnel and the inner wall of the flask were rinsed using deionized water. The flask was then heated again at 210 °C till clouds of white smoke were produced and the solution volume was reduced to about 2 mL. After cooling to room temperature, 5 mL of hydrochloride acid (AR) was added into the flask, which was then shaken well and covered with a plastic film. The solution was diluted to 25 mL the next morning to be analyzed by hydride generation-atomic fluorescence spectrometry (HG-AFS).

Chinese national standard reference material GSV-2 (100–140 μg Se kg^−1^) was chosen as standard sample with Relative Standard Deviation (RSD) ≤ 5%.

### 2.5. Measurement of Hemolymph Supernatant Antioxidant Enzyme Activities

Activities of hemolymph supernatant enzymes including alkaline phosphatase (AKP) (Disodium phenyl phosphate-4-Aminoantipyrine-Potassium ferricyanide method), lysozyme (LZM) (Lysozyme assay kit-nephelometry method), glutathione peroxidase (GPx) (5,5′-dithiobis-(2-nitrobenzoic acid) method (DTNB)), and superoxide dismutase (SOD) (hydroxylamine method) were measured using a UV/visible-6100 spectrophotometer (Shanghai Precision Instrument Co., Ltd., Shanghai, China) as described by the manufacturer’s protocols from the Institute of Biological Engineering of Nanjing Jiancheng (Nanjing, China). Three replicates for each sample were analyzed.

### 2.6. Analysis of Amino Acids, Protein, and Crude Fat

Amino acids, protein, and crude fats in the crabmeat samples were analyzed by the Shanghai Academy of Agricultural Sciences (Shanghai, China). Types and levels of all free amino acids in the sample were analyzed using an automatic amino acid analyzer (pH 5–5.5, 100 °C, 10–15 min). Protein content was determined by a semiautomatic nitrogen determination apparatus following the method GB/T 5009.5-2010. The soxhlet extraction method was applied according to the method GB/T 14772-2008 to measure the crude fat content. The difference between the two results obtained from the same sample was no more than 5% of the average of the two values.

### 2.7. Data Analysis

The results are expressed as mean value ± standard errors (x ± SE). The statistical analysis was performed using the procedure of two-sample *t*-test in Origin7.0. Statistically significant difference was reported when the probability of the result assuming the null hypothesis (*p*) is less than 0.05.

## 3. Results

### 3.1. Effects of Se-Biofortified Corn on the Total Se Level in Crabmeat

**Figure 1:**
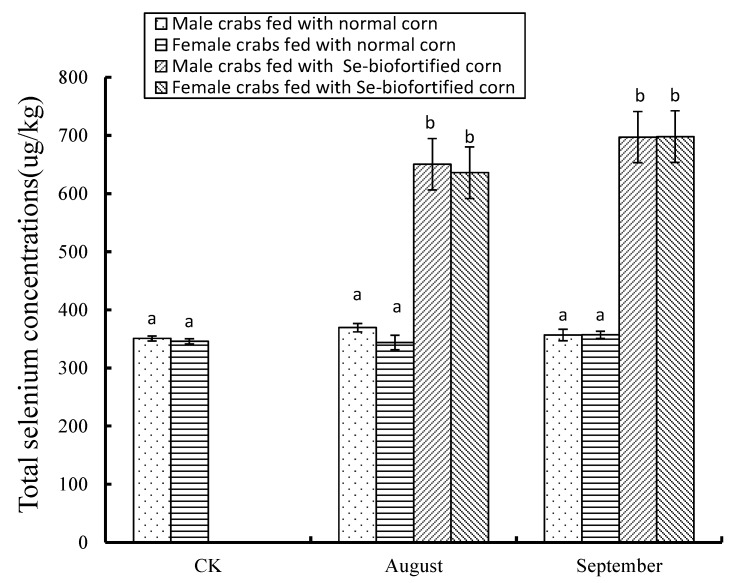
The total selenium levels in crabmeat samples. Note: Data of columns marked with the same letter are not statistically different (*p* > 0.05). CK: samples on 29 July 2015 right after the fourth shelling; August: samples on 30 August 2015 right after the fifth shelling; September: samples on 30 September 2015 at the harvest stage.

The total Se levels in crabmeat samples are shown in Figure 1. There were no significant differences between the male (350.8 ± 4.2 μg·kg^−1^) and the female crabs (345.8 ± 4.2 μg·kg^−1^) in the CK samples (*p* > 0.05). The one-month feeding of Se-biofortified corn increased the total Se levels to 650.9 ± 44.1 μg·kg^−1^ (male) and 635.9 ± 44.5 μg·kg^−1^ (female), which were increased 1.86-fold (male) and 1.84-fold (female) compared to those of the CK samples, respectively (*p* < 0.05). The total Se levels in crabmeat samples taken from the crabs fed normal corn for one month were 369.4 ± 7.2 μg·kg^−1^ (male) and 343.9 ± 12.4 μg·kg^−1^ (female), which were not significantly different than those of the CK samples (*p* > 0.05).

The two-month feeding of corn increased the total Se levels to 697.1 ± 43.4 μg·kg^−1^ (Se-biofortified corn, male), 356.7 ± 2.79 μg·kg^−1^ (normal corn, male), 697.8 ± 5.6 μg·kg^−1^ (Se-biofortified corn, female), and 356.9 ± 2.53 μg·kg^−1^ (normal corn, female), which were similar to those of the one-month feeding (*p* > 0.05). The results suggested that the feeding of Se-biofortified corn for one month could significantly increase the total Se level in crab (*p* < 0.05), which then kept at a state of balance. No difference in the total Se levels was found between the male and the female crabs (*p* > 0.05).

### 3.2. Effects of Se-Biofortified Corn on Hemolymph Supernatant Antioxidant Enzyme Activities

**Figure 2:**
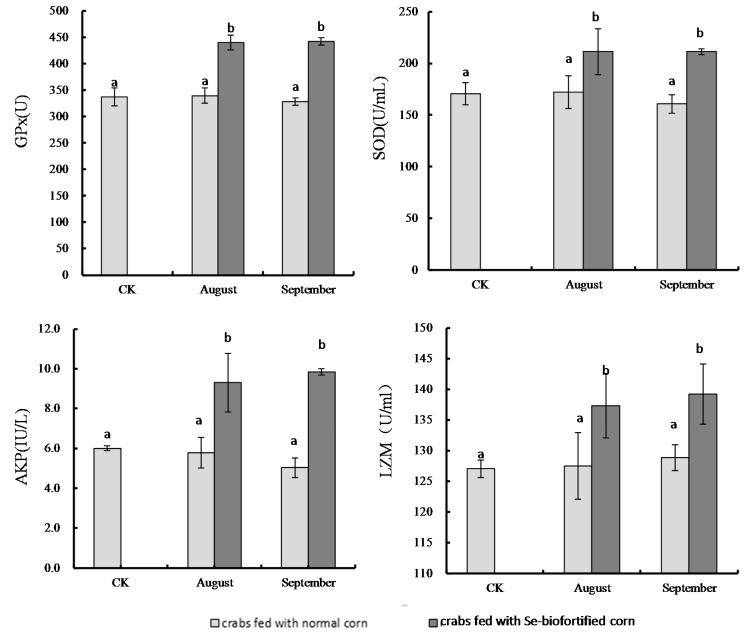
Activities of hemolymph supernatant antioxidant enzymes. Note: Data of columns marked with the same letter are not statistically different (*p* > 0.05). CK: samples on 29 July 2015 right after the fourth shelling; August: samples on 30 August 2015 right after the fifth shelling; September: samples on 30 September 2015 at the harvest stage.

Supernatant antioxidant enzyme activities are shown in Figure 2. Activities of GPx, SOD, LZM, and AKP in the fourth molt samples were 337.3 ± 14.5 U, 170.7 ± 15.9 U·mL^−1^, 127.0 ± 15.4 U·mL^−1^, and 6.00 ± 0.8 IU·100 mL^−1^, respectively. The one-month feeding of Se-biofortified corn increased the enzyme activities compared to the CK sample 1.30-fold (to 440.2 ± 13.6 U, GPx), 1.24-fold (to 211.4 ± 22.3 U·mL^−1^, SOD), 1.08-fold (to 137.3 ± 10.3 U·mL^−1^, LZM), and 1.55-fold (to 9.31 ± 1.5 IU·100 mL ^−1^, AKP), respectively (*p* < 0.05). Supernatant antioxidant enzyme activities in crabmeat samples taken from the crabs fed normal corn for one month were 339.6 ± 14.5 U (GPx), 172.3 ± 15.9 U·mL^−1^ (SOD), 127.5 ± 5.4 U·mL^−1^ (LZM), and 5.78 ± 0.8 IU·100 mL^−1^ (AKP), which were not significantly different than those of the CK sample (*p* > 0.05).

The two-month feeding of corn did not result in any difference in hemolymph supernatant antioxidant enzyme activities compared to the one-month feeding, with levels of 328.7 ± 7.1 U (GPx), 160.7 ± 8.94 U·mL^−1^ (SOD), 128.8 ± 2.1 U·mL^−1^ (LZM), and 5.03 ± 0.49 IU·100 mL^−1^ (AKP) for the normal corn-fed crabs, and 342.5 ± 7.0 U (GPx), 211.3 ± 2.6 U·mL^−1^ (SOD), 139.2 ± 4.9 U·mL^−1^ (LZM), and 9.85 ± 0.2 IU·100 mL^−1^ (AKP) for the crabs fed Se-biofortified corn.

### 3.3. Effects of Se-Biofortified Corn on the Contents of Protein, Crude Fats, and Amino Acids in Crabmeat

Table 1 shows the protein contents, crude fat levels, and the types and levels of amino acids in the samples. The protein content in crabmeat of the crabs fed Se-biofortified corn for one month was found to be 8.22 g·kg^−1^, which was significantly higher than that of the crabs in the CK sample (7.81 g·kg^−1^) (*p* < 0.05), and significantly higher than that of the crabs fed normal corn for one month (8.00 g·kg^−1^) (*p* < 0.05). The next one-month feeding of Se-biofortified corn resulted in the continuous increase of the protein content in crabmeat to 8.38 g·kg^−1^. However, the protein content in crabmeat would significantly decrease to 7.36 g·kg^−1^ after continuous feeding with normal corns.

The crude fat levels in all sample groups were not significantly different in the first month of feeding with corn (*p* > 0.05). The second month of feeding with Se-biofortified corn, however, significantly decreased the crude fat level in crabmeat (0.21 g·kg^−1^) compared to that from the crabs fed a diet of normal corn (0.23 g·kg^−1^) (*p* < 0.05).

In total, 16 types of amino acid were determined in the present study, among which lysine (Lys), phenylalanine (Phe), methionine (Met), threonine (Thr), isoleucine (Ile), leucine (Leu), and valine (Val) are essential amino acids (EAAs). Half essential amino acids (HEAAs) include histidine (His) and arginine (Arg). Glutamic acid (Glu), glycine (Gly), alanine (Ala), aspartate (Asp), tyrosine (Tyr), proline (Pro), and serine (Ser) are non-essential amino acids (NEAAs). Phe, Glu, Gly, Ala, Asp, and Tyr are grouped into delicious amino acids (DAAs) [20,21]. It was found that at the harvest, the ∑TAAs, ∑HEAAs, and ∑NEAAs in crabs fed Se-biofortified corn were similar to those in crabs of the CK sample, whereas those in the crabs fed normal corn decreased significantly from 6.96 g·kg^−1^ to 6.38 g·kg^−1^, 0.98 g·kg^−1^ to 0.88 g·kg^−1^, and 3.44 g·kg^−1^ to 3.22 g·kg^−1^, respectively (*p* < 0.05). The total levels of EAAs (∑EAAs) in crabs fed either Se-biofortified corn or normal corn were similar to those in crabs of the CK sample after the one-month feeding (*p* > 0.05). The two-month feeding of Se-biofortified corn, however, significantly enhanced the total level of EAAs (∑EAAs) in the crabs to 3.87 g·kg^−1^, which was increased 1.27-fold compared to that in the crabs fed normal corn (3.06 g·kg^−1^), and increased 1.52-fold compared to that in the crabs of the CK sample (2.55 g·kg^−1^) (*p* < 0.05). The total level of DAAs (∑DAAs) in the crabs fed normal corn significantly decreased with the feeding duration from 3.40 g·kg^−1^ (the CK sample) to 3.13 g·kg^−1^ (the August sample) (*p* < 0.05), and then to 2.85 g·kg^−1^ (the September sample), whereas the total levels of DAAs (∑DAAs) in the crabs fed Se-biofortified corn were 3.15 g·kg^−1^ (the August sample) and 3.15 g·kg^−1^ (the September sample), which were not significantly different from those in the crabs of the CK sample (*p* > 0.05).

## 4. Discussion

**Figure 3:**
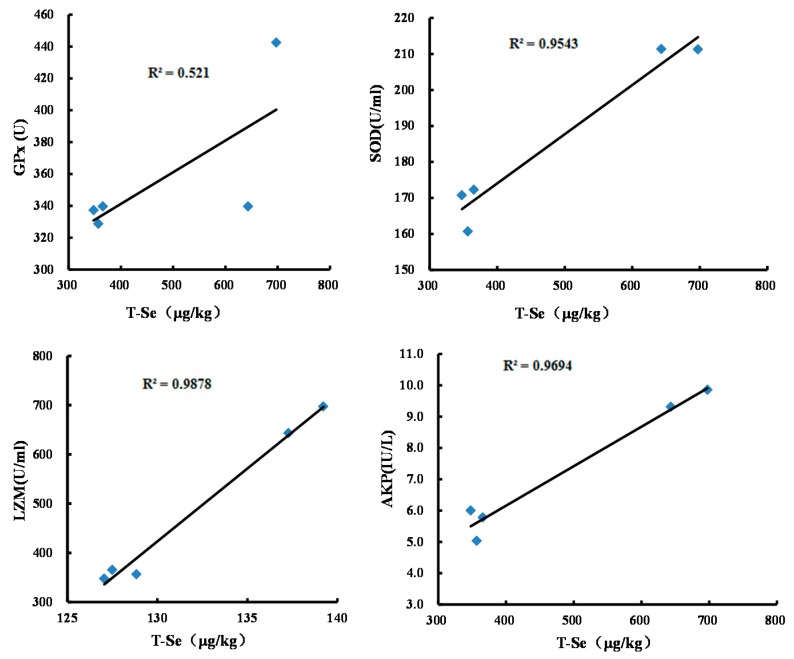
The correlation of the total Se levels in crabmeats and the activities of the hemolymph supernatant enzymes.

Evaluation of the hemolymph supernatant biochemical characteristics has become an important method in studying the normal physiology, pathological process, and toxicological mechanism of organisms [22,23,24]. Free radicals in healthy organisms have been proved to be related to lipid peroxidation activity [25]. Antioxidant enzymes in the hemolymph supernatant can eliminate free radicals, e.g., GPx helps to remove H_2_O_2_ and lipid peroxide, and SOD is a scavenger of O_2_^−^. Some hemolymph supernatant enzymes are helpful in enhancing the immunity and well-being of organisms, e.g., LZM can eliminate pathogenic bacteria, and AKP, as a nonspecific phosphomonoesterase, helps in the absorption of dissolved calcium by crustaceans and the formation of calcium phosphate. AKP can also catalyze the hydrolysis of phosphate monoester and the transfer of phosphoric acid [26]. Figure 3 shows that in this study, the total Se levels in crabmeat are positively correlated with the contents of hemolymph supernatant GPx (*r* = 0.87, *p* < 0.01), SOD (*r* = 0.88, *p* < 0.01), LZM (*r* = 0.70, *p* < 0.01), and AKP (*r* = 0.85, *p* < 0.01), suggesting that the intake of Se-enriched corn significantly improve the anti-oxidative capability as well as the immunity of Chinese mitten crab (*p* < 0.05).

GPx is an antioxidant selenoenzyme, functioning in removing free radicals and protecting cells [27]. Wang et al. (2007) proved that Na_2_SeO_3_ and SeMet could significantly enhance the activity of hemolymph supernatant GPx in crucian carp (*Carassius auratus gibelio*) [28]. Qin et al. (2016) demonstrated the ability of nano-Se in increasing the activity of GPx in Chinese mitten crab (*Eriocheir sinensis*) [9]. Wang et al. (2009) found that the hemolymph supernatant SOD activity in shrimp (*Neocaridinaheteropoda*) fed Se was significantly higher than those fed the supplemented control diet [29]. It has been suggested that the change of GPx/SOD ratio indicated the change in anti-oxidative status in organisms [30]. In the present study, the GPx/SOD ratio before the feeding of Se was 1.86 ± 0.25. After the one-month feeding of Se-biofortified corn, the ratio of GPx/SOD increased from 1.92 ± 0.02 (the CK sample) to 2.10 ± 0.08 (*p* < 0.05). The two-month feeding of Se-biofortified corn resulted in a GPx/SOD ratio of 2.11 ± 0.02, which was significantly higher than that of 1.95 ± 0.03 in the crabs fed normal corn (*p* < 0.05). The results suggested the anti-oxidative capability of Chinese mitten crab was enhanced by feeding with Se-biofortified corn.

As one of the non-specific immune factors, LZM functions in the inhibition of Gram-positive bacteria. It was found the concentration of hemolymph supernatant LZM in blue gourami (*Trichogaster trichopterus*) was 33.72% higher after two weeks of exposure to sodium selenite [31]. Liu et al. (2013) suggested that the LZM activity in *Litopenaeusvannamei* could be enhanced by the combined addition of βG and Se [32]. LZM activity was also significantly affected by dietary Se on yellowtail kingfish (*Seriola lalandi*) [33].

The increase of the AKP activity can provide more inorganic phosphorus to accelerate the phosphorylation of ADP to produce ATP, thus accumulating energy to promote growth and enhancing the non-specific immunity of organisms [34]. AKP helps in the absorption of dissolved Ca, the synthesis of calcium phosphate, and the formation and secretion of chitin in Chinese mitten crab. Wei et al. (1995) found that the effect of Se on the AKP activity was negligible, but Se could mitigate the inhibitory effect of copper on the AKP activity in red sea bream (*Chrysophrys major*) [35]. Regoli and Principato (1995) suggested that the increase of AKP activity in mussel, *Mytilus galloprovincialis*, was not significantly related to the increase of Se levels, but might be due to adaptation or compensatory mechanisms during the chronic exposure to a polluted environment [36,37,38].

**Figure 4:**
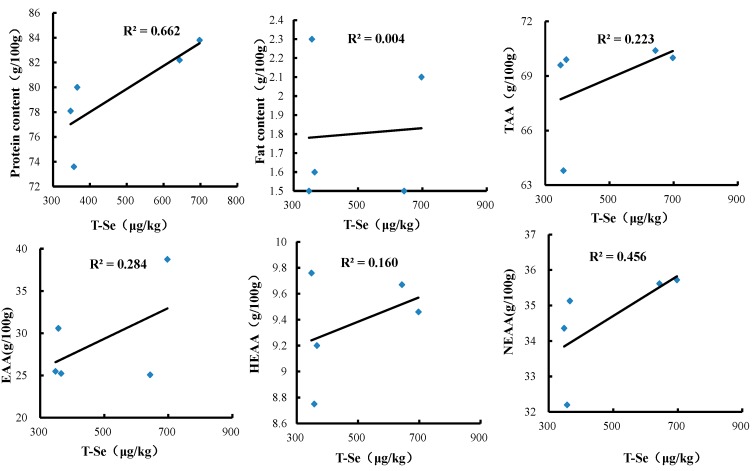
The correlation of the total Se levels in crabmeat and the protein contents, the fat levels, and the amino acid levels.

Figure 4 shows the significantly positive correlations that were observed between the total Se levels in crabmeat and the protein contents (*r* = 0.81, *p* < 0.01), the ∑EAA levels (*r* = 0.53, *p* < 0.01), and the ∑NEAA levels (*r* = 0.68, *p* < 0.01). Se levels were not associated with the crude fat levels (*r* = 0.07, *p* < 0.01) or the ∑DAA contents (*r* = 0.05, *p* < 0.01), and positively but not significantly correlated with the ∑TAA contents (*r* = 0.47, *p* < 0.01) and the ∑HEAA contents (*r* = 0.40, *p* < 0.01).

Se, as selenocysteine in 5′-deiodinase, can catalyze the conversion of tetraiodothyronine (T_4_) into triiodothyronine (T_3_). T_3_ controls the synthesis of growth hormone as well as the improvement of insulin levels, and promotes protein synthesis in muscle and the growth of animals [39]. A study conducted by Hicks et al. (1984) suggested that Na_2_SeO_3_ was not associated with the protein contents in rainbow trout (*Salmogairdneri*) [40]. Li et al. (2016), however, studied the effect of dietary Se sources on growth, body composition, and anti-oxidant performance of *Litopenaeusvannamei*, and found that Se could significantly increase body crude protein contents and decrease crude fat contents [41]. SeMet, as an organic Se source, can be incorporated into protein synthesis in place of methionine [42].

Up to now, few studies have been conducted to evaluate the effects of Se on muscle amino acids in Chinese mitten crab. A previous study revealed that nano-Se could increase the contents of free amino acids in Chinese mitten crab (*Eriocheir sinensis*) [43]. An ideal protein source contains about 40.0% EAAs according to Food and Agriculture Organization/ World Health Organization (FAO/WHO) recommendation. In this study, the ratio of EAAs to total proteins was 36.62% before feeding Se-biofortified corn. After one month of feeding with Se-biofortified corn, the ratios of EAAs to total protein were 36.11% and 35.62% for the fifth normal group and the fifth Se group, respectively. Two months of feeding with Se-biofortified corn significantly increased the ratio of EAAs to total protein for the harvest Se group to 55.34%, which was 7.41% higher than that for the harvest normal group (47.93%, *p* < 0.05) and 18.72% higher than that of the fourth molt sample (*p* < 0.05). The protein contents, the levels of amino acids, and the crude fat levels in Chinese mitten crab fed Se-biofortified corn were significantly higher than those in the CK samples. The present study demonstrated that Se-biofortified corn could improve the contents of amino acids as well as promote protein synthesis and the accumulation of crude fat in Chinese mitten crab. These findings are consistent with those of Ashouri et al. (2015) on Cyprinus carpio (*Cyprinus carpio*) [44] and Tian et al. (2014) on juvenile Chinese mitten crabs (*Eriocheir sinensis*) [45].

## 5. Conclusions

The total Se contents in the muscle tissue of Chinese mitten crabs fed dietary Se-biofortified corn (with SeMet as the major Se-containing compound) were in the range of 635.9–697.8 μg·kg^−1^, which were significantly higher than those of Chinese mitten crab fed dietary normal corn (350.8–360.0 μg·kg^−1^). The diet of Se-biofortified corn also improved the activities of hemolymph supernatant enzymes including GPx, SOD, LZM, and AKP, which were increased 1.04-fold, 1.31-fold, 1.08-fold, and 1.96-fold, respectively, compared to those of crabs fed normal corn, indicating that the Se-biofortified corn diet could enhance the anti-oxidative capacity of Chinese mitten crab. The contents of protein and amino acids in the muscle tissue of Chinese mitten crab fed Se-biofortified corn were also 7.29% and 26.69% higher than those of Chinese mitten crab fed normal corn, whereas the levels of crude fat in the former were significantly decreased from 0.23 g·kg^−1^, as determined in the latter, to 0.21 g·kg^−1^. The present study demonstrated that feeding with Se-biofortified grain, e.g., Se-biofortified corn, might be an effective strategy to enhance Se intake levels in aquatic animals, and to improve their anti-oxidative capacity.

## Figures and Tables

**Table 1:** Effects of Se-biofortified corn on the contents of protein, crude fats, and amino acids in crabmeat.

Item	July Sample (CK)	August Sample		September Sample	
		Normal Corn	Se-Biofortified Corn	Normal Corn	Se-Biofortified Corn
	g·kg^−1^				
Protein	7.81	8.00	8.22	7.36	8.38
Crude fat	0.15	0.16	0.15	0.23	0.21
Lys ^1^	0.54	0.55	0.54	0.49	0.54
Phe ^1,3^	0.35	0.32	0.32	0.29	0.32
Mer ^1^	0.21	0.21	0.21	0.21	0.22
Thr ^1^	0.36	0.36	0.35	0.32	0.35
Ile ^1^	0.26	0.25	0.25	0.23	0.24
Leu ^1^	0.57	0.57	0.57	0.5.2	0.56
Val ^1^	0.27	0.26	0.26	0.23	0.25
His ^2^	0.24	0.23	0.23	0.21	0.23
Arg ^2^	0.74	0.69	0.74	0.67	0.72
Glu ^3,4^	1.07	1.10	1.09	1.01	1.08
Gly ^3,4^	0.33	0.39	0.42	0.33	0.42
Ala ^3,4^	0.57	0.59	0.60	0.55	0.62
Asp ^3,4^	0.70	0.70	0.69	0.64	0.6.9
Tyr ^3,4^	0.38	0.36	0.35	0.32	0.35
Pro ^4^	0.09	0.19	0.11	0.09	0.12
Ser ^4^	0.30	0.3	0.31	0.28	0.30
∑EAA	2.55	2.52	2.51	3.06	3.87
∑HEAA	0.98	0.92	0.97	0.88	0.95
∑NEAA	3.44	3.51	3.56	3.22	3.57
∑DAA	3.40	3.13	3.15	2.85	3.15
∑TAA	6.96	7.00	7.04	6.38	0.70

Note: The amino acids marked with “1” are essential amino acids (EAA), with “2” are half essential amino acids (HEAA), with “3” are delicious amino acids (DAA), and with “4” are non-essential amino acids (NEAA); ∑ represents the sum of amino acids. TAA denotes total amino acids.
